# Analysing Dynamical Behavior of Cellular Networks via Stochastic Bifurcations

**DOI:** 10.1371/journal.pone.0019696

**Published:** 2011-05-25

**Authors:** Anna Zakharova, Jürgen Kurths, Tatyana Vadivasova, Aneta Koseska

**Affiliations:** 1 Center for Dynamics of Complex Systems, University of Potsdam, Potsdam, Germany; 2 Potsdam Institute for Climate Impact Research, Potsdam, Germany; 3 Institute of Physics, Humboldt University Berlin, Berlin, Germany; 4 Saratov State University, Saratov, Russia; Keio University, Japan

## Abstract

The dynamical structure of genetic networks determines the occurrence of various biological mechanisms, such as cellular differentiation. However, the question of how cellular diversity evolves in relation to the inherent stochasticity and intercellular communication remains still to be understood. Here, we define a concept of stochastic bifurcations suitable to investigate the dynamical structure of genetic networks, and show that under stochastic influence, the expression of given proteins of interest is defined via the probability distribution of the phase variable, representing one of the genes constituting the system. Moreover, we show that under changing stochastic conditions, the probabilities of expressing certain concentration values are different, leading to different functionality of the cells, and thus to differentiation of the cells in the various types.

## Introduction

Cellular responses to external stimuli characterize structural and functional cellular processes. These responses on the other hand are determined by the dynamics of the underlying genetic networks. Therefore, the understanding of their dynamical characteristics can be used to generate novel insight into the properties of gene regulatory networks. In dynamical systems theory, the investigations how system-level behavior changes as a function of particular parameter, using a given deterministic model, is subject of bifurcation analysis. Determining mathematically the existence, number and stability of distinct attractors (steady states and limit cycles) provides a detailed and comprehensive picture of the dynamical structure of the system, from which various functional properties can be further extrapolated.

It is known, for e.g., that cells in multicellular organisms switch between distinct cell fates, such as proliferation or differentiation in specialized cell types. Theoretical studies of complex networks suggest that they can exhibit organized dynamics, e.g. have a number of stable attractors in which large fractions of the genes exhibit identical behavior (steady or oscillatory), despite their global interdependence. This raises the possibility that attractor states represent various cell types [1]–[5], or more generally, different cell fates [6], [7].

However, this approach is too idealistic because cellular networks are inherently noisy. It was shown that noise can shift or join basins of attraction, i.e. straightforward correspondence between deterministic attractors and their stochastic counterparts can not be established. Therefore, we generalize here the deterministic systems theory to stochastic dynamical systems and investigate the complexity of genetic networks' behavior in terms of stochastic bifurcations. In general, stochastic bifurcations are characterized with a qualitative change of the stationary probability distribution, e.g., a transition from unimodal to bimodal distribution [8]–[11]. Such a change in the distribution results in a change of other stochastic characteristics of the system that can be observed experimentally as well (e.g. variances, correlation functions and power spectra of the oscillations). Additionally, stochastic bifurcations can also become apparent through a change of stability of trajectories belonging to a certain set with a given invariant measure [12]. The first type of stochastic bifurcations is called 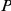-bifurcations (phenomenological bifurcations), whereas the second one, 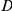-bifurcations (dynamical bifurcations) [13]. Moreover, stochastic bifurcations can also consist of two steps: 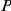-bifurcations and 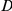-bifurcations, separated in the parameter space by a certain bifurcation interval. Thus, this approach generally provides a qualitative description of the system's behavior, both, when estimating its bifurcation structure from noisy experimental data or when analyzing it using a given mathematical model.

Here, we address the question how (large) complex networks can give rise to various coherent responses (associated with deterministic stable attractors) under stochastic influence. In particular, we study the qualitative transformation of the distribution of the phase variables of identical genetic oscillators which constitute the synthetic genetic network under consideration. In the deterministic limit, the network displays multistable behavior, which in turn contributes to the complex behavior which is observed in the stochastic case. Additionally, in order to explain the ubiquitous nature of cellular diversity in multicellular organisms, it is important to understand how the reaction dynamics allowing for cell differentiation evolves in relation to the intercellular dynamics of the network and the inherent stochasticity. Thus, using the concept of stochastic bifurcations and adopting a dynamical systems model, we propose a description for terminal cell fate in a stochastic environment. In particular, we consider the dynamics of cellular network to be determined by two separate factors: *i)* stochastic and *ii)* external factor. Under stochastic factor we understand the presence of random fluctuations which can arise from infrequent molecular events involving small number of molecules. Additionally, the presence of stochastic fluctuations in genetic networks can be regarded as a “survival factor”. It is known e.g., that a sufficient amount of noise can induce jumps between coexisting states in the network [14]. This can be interpreted as an ability of the system to adapt to changing environmental conditions. In addition to the stochastic factors, the dynamics of genetic networks is very often determined via the influence of external factors, such as external environmental signals or a (co)regulating network in the system. Hence, we investigate separately how the influence of these factors is reflected in the dynamical structure of genetic networks and describe the stochastic bifurcation transitions which occur under the given system's conditions.

## Methods

### Structure of the Model

Recently, the study of complex biological networks has profited from the notion of reduced complexity which synthetic biology offers. In particular, the design of artificial genetic units resembling submodules of natural circuitry (e.g. switches [15], [16] and oscillators [17]–[20]) on the one hand offers the opportunity to study specific cellular functions and signaling pathways for which limitations occur in the natural environment, and on the other hand, it allows to investigate synthetic systems for improvement or regulation of given biological properties. Thus, we consider here a model proposed in Ref. [21], that describes a population of synthetic gene relaxation oscillators coupled via intercellular signaling mechanism, known as quorum sensing mechanism. The usage of oscillating units in the study of dynamical properties of genetic networks in general is of significant importance, since a vast range of proteins that govern fundamental physiological processes, such as insulin secretion [22], cell cycle and circadian rhythms [23], [24] display oscillatory behavior.

**Figure 1:**
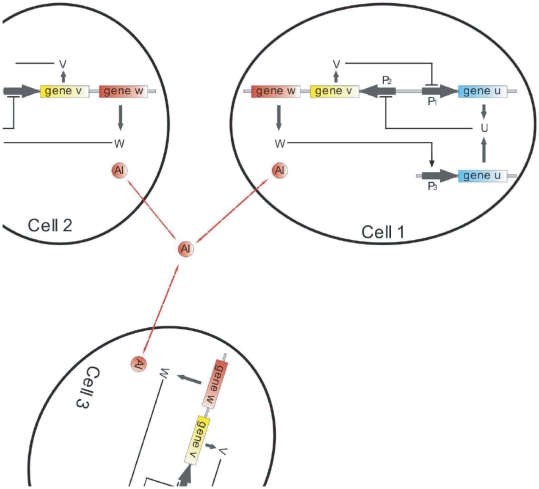
A simplified scheme of the genetic network under investigation. Mutually repressing genes 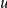 and 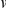 form the toggle switch inside separate cells. The 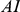 molecule denoted as 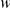, diffuses through the membrane, providing intercell coupling.

The underlying genetic circuit (Fig. 1) contains a toggle switch composed of two genes, *lacI*, denoted here as *u*, and *cI857* (*v*), that inhibit each other by repressing transcription from their respective promoters P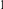 and P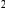. This circuit is known to lead to bistable behavior [15]. The promoter P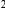 also drives the expression of a third gene, *luxI* (*w*) that synthesizes a small autoinducer (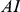) molecule, which is able to diffuse in and out of the cell. The 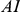 activates transcription of promoter P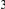. Placing a second copy of the *u* gene under the control of this promoter provides both an additional feedback loop to the toggle switch, and a mechanism that couples the switch to all cells in the population via quorum sensing.

The time evolution of the proteins involved in the genetic circuit represented in Fig. 1 can be described by the following dimensionless equations:
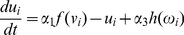
(1)

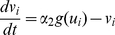
(2)

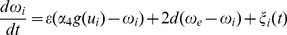
(3)

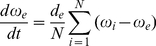
(4)where the subindex 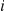 denotes the cell index, with 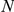 being the total number of cells. The activity of the promoters P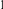, P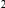 and P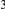 are described by the Hill functions 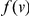, 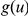 and 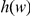, respectively, defined as:
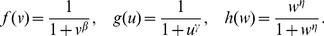
(5)


The parameters 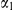 and 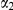 determine the expression strength of the toggle switch genes, while 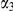 represents the activation of 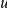 from promoter P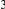. The expression of the lux gene 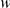 is measured by the parameter 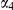. 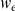 stands for the extracellular and 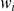 for the intracellular concentration of 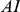. Time has been rescaled by the lifetime of 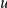 and 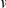, assumed to be equal. The parameter 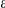 measures the ratio between the lifetimes of the toggle-switch genes and the 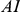 and is assumed to be small. This separates the dynamics of the cells into two very different time scales: with fast dynamics of 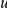, 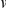 and 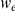 and slow dynamics of 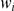. Thus, the parameter 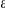 controls the stiffness of the oscillator and therefore it has a constant, nonzero value, which we fix here at 0.01. The dynamics of the 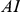 (investigated in detail in [21]) introduces an additional feedback loop into the toggle switch and can lead to oscillatory behavior even in isolated cells [21]. The coupling coefficients 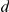 and 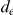 depend mainly on the diffusion of the 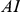 through the cell membrane. We assume that the experiments are carried out in a continuously stirred, constant volume flow reactor. The extracellular medium is homogeneous and the number of cells is kept constant by continuous dilution of the cell culture by the steady inflow of fresh growth medium and outflow of extracellular medium and cells.

One can biologically manipulate the relevant parameters by controlling e.g. the number of plasmids per cell, protein decay rate or 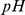 of the solution etc., which enables experimental control of the circuits dynamics. The stochastic factor is represented using an additive noise source 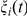, which is a Gaussian white noise with zero mean and correlation given by 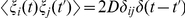, where 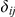 is the Kronecker delta, 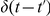 is the Dirac function and 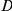 is the constant that characterizes the noise intensity.

The corresponding stochastic differential equations are of Langevin type. The numerical integration is done using the scheme, based on the Euler – Maruyama difference approximation and correction according to the Heun method [25], [26].

We note here that a multiplicative noise source does not change qualitatively the results presented in this work (results not shown). Furthermore, we model the influence of various external factors in its most simplified sense, using external harmonic forcing. In this case, the Eq. 1 is substituted with
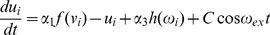
(6)Here, 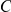 represents the amplitude of the external force, whereas 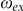 describes its frequency.

## Results

### Dynamical behavior of a single cell

#### Stochastic bifurcation structure

**Figure 2:**
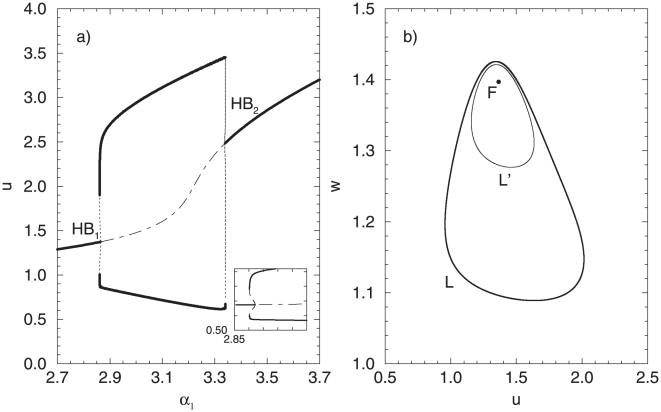
Characteristics of a single oscillator in the deterministic case (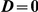). (A) Bifurcation diagram. Solid lines correspond to stable, and dashed lines to unstable solutions. The dash-dotted line indicates the unstable focus. (B) Phase portrait for the bistability region. 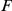 denotes the stable focus, 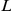 and 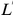 – the stable and unstable limit cycles, correspondingly. Unless noted differently, the parameters are defined as follows: 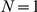, 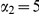, 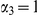, 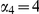, 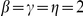, 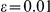 and (B) 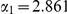.

The dynamical structure of cellular networks is influenced by the characteristics of its constituent parts (distinct cells), whose behavior however displays changes when switching from deterministic to stochastic dynamical systems representations. Therefore, we evaluate initially the dynamical changes which occur in a single cell especially when the expression strength of one of the genes (constituting the genetic oscillator), the parameter 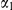, is changed. The resulting bifurcation diagram in Fig. 2a (Fig. 2a, zoomed region) shows that the system is characterized with bistability in the deterministic case: two co-existing attractors are present in the phase plane – a stable focus 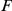 and a stable limit cycle 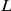 separated by the unstable limit cycle 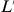 (the phase portrait for the bistability area is shown in Fig. 2b). Moreover, the bistability region is bounded by a tangent bifurcation from one side (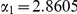) and a subcritical Hopf bifurcation (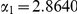) from the other one.

**Figure 3:**
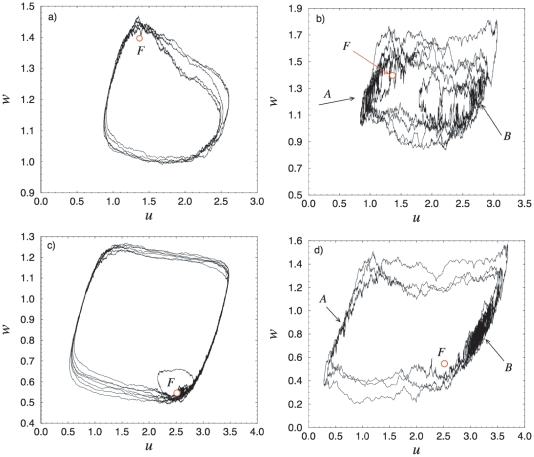
Phase portraits of a single oscillator for 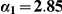 and different values of the noise intensity 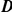: (A) 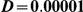 and (B) 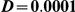 and for 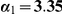 and (C) 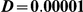 and (D) 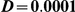. 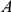
 and 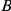 indicate the areas in which the stochastic trajectories spend most of the time.

However, the behavior of the cells in reality is not deterministic. This implies that under the influence of noise, the structure which we observed in the deterministic case will not be sustained. Figs. 3a–d demonstrate phase portraits of a single oscillator for different 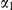 values and noise intensities 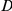. In particular, for small noise intensity (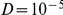), even for 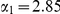, for which in the deterministic case there exist only one attractor, the stable focus 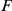, the system performs an oscillatory behavior, similar to the motions on the limit cycle, as shown in Fig. 3a (the phase portrait for the same noise intensity, but for 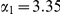 is shown in Fig. 3c). Therefore, we can state that the behavior of the system is very similar to those of excitable systems. The corresponding stochastic structure is characterized with one invariant set of trajectories in the phase space. For larger noise intensities (e.g. 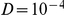) then, two main areas can be identified in the phase projections (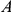 and 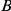 in Figs. 3b,d), where the stochastic trajectories spend dominant part of time. These areas correspond to the minimal (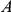) and maximal (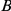) concentration levels of the expressed *LacI* protein - the upper and the lower branches of the limit cycle.

**Figure 4:**
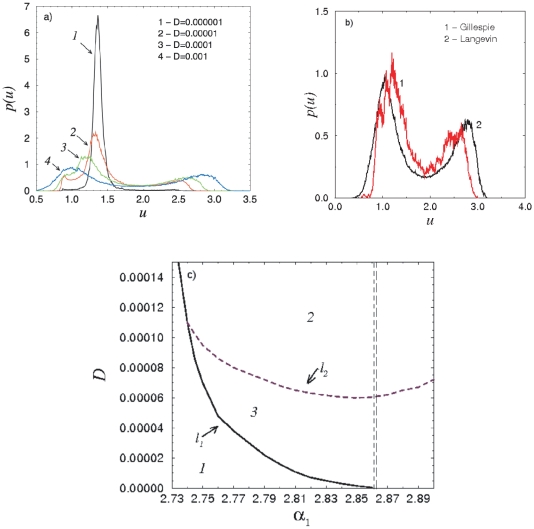
Stochastic bifurcations in a single oscillator. (A) Probability distributions 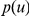 for 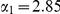 and different values of 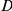 (indicated in the figure). (B) The comparison of probability distributions 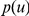 for the Gillespie algorithm (red curves) and Langevin equations (black curves) calculated for 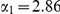 and 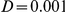. (C) Stochastic bifurcation diagram in the 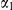
−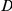 plane. The regions of the diagram are numerated according to the number of maxima of the probability distribution. Lines 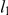 and 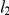 stand for the stochastic bifurcations. The dashed vertical lines correspond to a tangent bifurcation (the left line) and the subcritical Hopf bifurcation (the right line) in the deterministic system.

In order to analyze in detail the stochastic behavior of the cellular system in terms of stochastic bifurcations, we look next at the qualitative changes of the stationary probability distributions of the protein concentrations 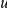. In the case of a single oscillator and for small noise intensities, the trajectories spend most of the time in the vicinity of the focus (which is the only stable solution in the deterministic case), and the distribution has one maximum (curve 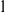 in Fig. 4a). A subsequent increase of the noise intensity 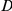 results in more frequent visits of the phase trajectory to regions away from the origin. In other words, the noise induces oscillations in the vicinity of the cycle, manifested with the appearance of two additional maxima in the distribution (corresponding to the upper and lower 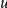 values of the cycle). Hence, the distribution evolves with 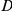. For 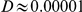 a transition from unimodal to multimodal distribution (curve 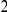 in Fig. 4a) occurs, i.e. the first stochastic P-bifurcation takes place. In the case of stronger noise intensity, however, the trajectory finds itself very rarely in the vicinity of the stable focus. Thus, the maximum corresponding to the focus decreases and shifts to the left, and for 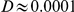 it merges with one of the maxima of the cycle and disappears. Here the second stochastic bifurcation occurs, i.e. we observe a transition from a multimodal to a bimodal distribution (curves 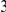 and 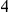 in Fig. 4a). Hence, the trajectory oscillates in the area where in the noiseless case the cycle is located.

In contrast to the paradigmatic mathematical model we have considered in our previous investigations [27], it is important to note here that in the case of genetic synthetic units, we observe qualitative transformation of the distribution of the phase variables themselves (not the distribution of the amplitude). The peculiarities of this probability distribution characteristics in the presence of noise are then interrelated with the deterministic location of the attractors in the model considered. In particular, the focus in the phase space is situated very close to the one side of the noise-induced stochastic cycle (see phase portrait for the noiseless case in Fig. 2(b), and the phase portraits in the presence of noise in Figs. 3a–d). For 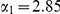, the focus is located on the left side of the stochastic cycle, close to the area 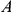 (Figs. 3a,b), where the probability distribution 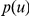 has a maximum. For 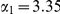, the focus shifts to the right side of the cycle and is close to the area 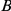 (Figs. 3c,d). Thus, the contribution of the oscillations with larger amplitude grows as the noise intensity 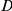 increases. Therefore, we observe a shifting of the peak corresponding to the focus to the direction of the cycle: for 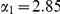, the peak shifts to the left, as shown in Fig. 4a (for 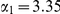, the peak shifts to the right - results not shown). The further increase of the noise intensity 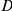 causes the disappearance of this peak, and consequently to the stochastic bifurcation. Due to the symmetry of the system, similar stochastic bifurcations take place near the 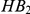. The difference is that the peak of the fixed point is shifted to the right, because in the deterministic case the focus is located close to the upper value of the cycle.

It is known that simulations of Langevin equations along the same lines as those considered here correlate well with a discrete description of the biochemical processes involved using, e.g., the Gillespie approach [28]. However, we have calculated additionally, using a simplied approach, the probability distributions obtained within a discrete description. For this purpose, we rewrote the stochastic model (1)–(4) in the Ito form i.e., introducing explicitly the Stratonovich drift term [29]. A typical probability distribution calculated with modified Gillespie algorithm as well as the distribution obtained using Langevin approach are shown in Fig. 4b. The results in both cases are qualititavly the same. Therefore, further on, we use the Langevin equations.

Additionally, the characteristic lines of the stochastic bifurcations which determine the transitions between different manifestations of the probability distributions are shown in Fig. 4c. The bifurcation diagram in the 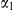
−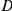 plane displays the distinct areas numerated according to the number of maxima (one, two or three) of the probability distribution. Thus, in region 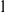 the distribution has one maximum, corresponding to the focus. It is bounded with the line 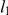 from the right side, characterizing the stochastic transitions to the region 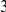, and the appearance of two additional maxima corresponding to the cycle - the distribution of the phase variable now has three peaks. Furthermore, on the line 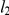 the central maximum disappears or interflows with the left maximum of the cycle, and the distribution becomes bimodal. Hence, the lines 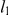 and 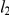 stand for the appearance respectively disappearance of maxima corresponding to the cycle and fixed point in the distribution 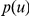 and, therefore, mark the stochastic P-bifurcations. We note here that these lines (particularly the line 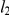) are plotted approximately, taking into account the complexity of the identification of the number of maxima in the numerical experiment. Moreover, it is complicated to understand what happens on the line 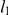, after it joins the line 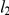. We suppose that the maximum of the fixed point coincides with the left maximum of the cycle and as a result, an increase of the right maximum of the cycle is observed.

In Fig. 4c, the dashed vertical lines correspond to a tangent bifurcation (the left line) and the subcritical Hopf bifurcation (the right line), which bound the bistability region in the deterministic case. On the other hand, the presence of the three-peak distribution (region 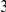 in Fig. 4c) in the stochastic case is similar to the bistability in the deterministic case, since the corresponding peaks for the focus and the limit cycle are observed in the distribution. However, the parameter area where region 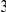 is observed in the stochastic case is significantly larger then its deterministic counterpart. We can infer that the analog of the bistability region significantly changes (increases) in the presence of noise.

It is important to note that even for 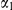 parmeter values for which in the deterministic system the cycle is located, we have observed in the stationary probability distribution, although slightly visible, a peak which is characteristic for the focus (results not shown here). Under stochastic influence, however, for the same 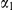 values this peak becomes significantly more pronounced. This is due to the fact that the focus is very close to the cycle in the phase plane, as it was mentioned before. Therefore, the rotation on the cycle is not uniform; when the trajectory approaches the focus, it slows down (decelerates). This allows for the generation of three-peak distributions for small noise intensities even for 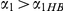, which characterizes the right-hand-side border of the deterministic bistability region.

As we have shown, even in the single-cell case, under stochastic influence, the levels of expressed protein concentration can vary with respect to the amount of noise present in the system. This means that the cellular genetic unit compensates for the fluctuation in the system by adapting the concentration levels to specific intervals, most profitable for the cell under given conditions (noise intensity present).

**Figure 5:**
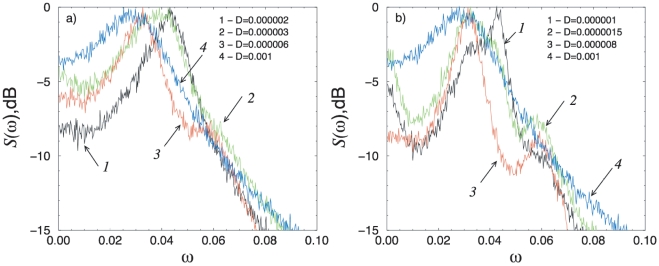
The normalized power spectra for a single oscillator and (A) 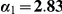, (B) 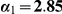 and different values of 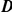 (indicated in the figure). The normalized spectrum is 
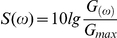
, where 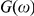 is the spectral power density of the oscillations 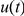 and 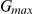 is its maximal value.

Previously, it was shown that for isochronous systems (the frequency of oscillations does not depend on their amplitude) near the subcritical Hopf bifurcation, an effect similar to coherence resonance (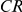) can be observed [27], [30]. The 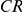 phenomenon, also known as autonomous stochastic resonance, shows that an optimal amount of noise enhances an intrinsic periodic behavior in stochastic nonlinear systems [31]. However, the model of the genetic circuit we consider here is rather anisochronous (i.e. the frequency depends on the amplitude of oscillations). Nevertheless, the degree of this dependence, or the difference between the frequencies of oscillations near the fixed point 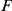 and far away from it, is rather small. This allows for a 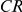-like effect to be observed here, manifested through a minimal width of the spectrum for intermediate noise intensities (Fig. 5a,b). These values of the noise intensity are further related to the 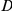 value at which the stochastic bifurcation takes place. In the current case, the 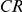-like effect is observed in the vicinity of the bottom part of line 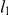, in the region to the left of the tangent bifurcation of the deterministic system. In particular, for 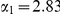 respectively 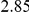 and small noise intensity (region 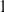 in Fig. 4c), the spectral maximum corresponds to the frequency of oscillations near the focus 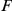 (curve 1 in Fig. 5a,b). As the contribution of the oscillations on the stochastic cycle grows with the increase of noise, a second spectral maximum at a lower frequency appears, corresponding to the oscillations on the stochastic cycle. Since the frequencies of these two maxima are close to each other, one wide spectral line is observed near the stochastic bifurcation line 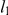 (curve 2 in Fig. 5a,b). Above this line (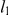), however, the spectral maximum is determined mainly by the oscillations on the cycle. Thus, near 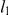 the spectrum narrows down (curves 3 in Fig. 5a,b). When the noise intensity is increased even further, then the spectrum becomes wider again (curves 4 in Fig. 5a,b). This means that an optimal value of the noise intensity exists, for which the spectrum of stochastic oscillations has minimal width: the effect of 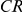 depends on the distance from 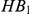 (distance from 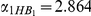). The more this value is approached, the 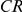-like effect becomes more pronounced (the 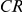-like effect is more evident for 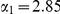 then for 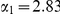, as shown in Fig. 5).

This 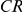-like effect which we demonstrate here shows that for specific, intermediate noise intensities, the genetic unit displays most ordered dynamical behavior, even under ongoing stochastic influence. We speculate that the noise which is inherently present in biological systems is profitable for the underlying networks, resulting in a coordinated dynamical behavior.

#### Cellular fate controlled by external signal

**Figure 6:**
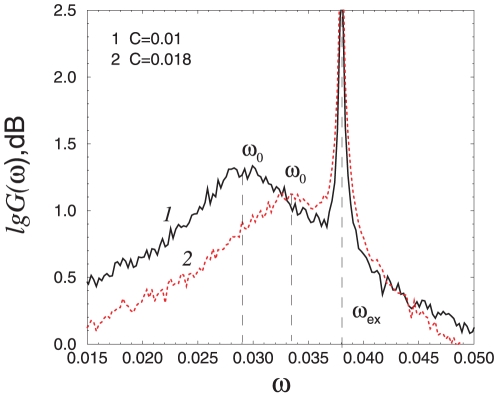
The power spectra for a single oscillator in the presence of noise and external harmonic forcing for 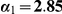, 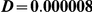 and different values of the external force amplitude 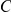 (indicated in the figure). The eigenfrequency is marked as 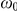. The frequency of the external forcing is 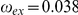. For convenience of the comparison of the results, the spectral power density 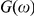 is not normalized here.

The expression of a particular protein of interest is in general determined via the influence of “external” factors, such as environmental signals or (co)regulating networks in the cellular organism. Hence, despite stochastic influence, the dynamics of the network is influenced also by external signals, which contribute to the regulation of the gene expression in the system. The form and the strength of interaction which leads to regulation of protein expression can vary from standard periodic signals (e.g. genes which are regulated via the circadian clock) to complex ones which integrate the outputs from various genetic and/or signaling networks. In what follows we investigate, in the most simplified form, the influence of external factors on protein production, if a periodic signal signal is considered under stochastic conditions. The system is now described by substituting Eq. 1 with Eq. 6. For a small amplitude of the external forcing and under very small noise (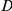 of order 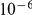), the spectrum becomes very wide, but it is possible to distinguish two separate frequencies, the eigenfrequency of the oscillator 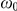 and the frequency of the external signal 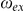. As already discussed, in the presence of noise, oscillations are induced in the system to the left of the tangent bifurcation. A further increase of the amplitude value results in shifting of the line of eigenoscillations towards the frequency of forcing (Fig. 6). The corresponding effect does not change when considering cellular networks of any size (results not shown). Thus, external factors influence strongly the expression of a particular gene, by modifying the intervals of expression of the particular protein. Additionally, the strength of interaction translated in the amplitude of the external signal influences the regulation of the protein expression, by adapting the eigenfrequency of protein expression.

### Estimating stochastic bifurcations of genetic networks

The dynamical structure of coupled cellular systems differs from the single cellular unit. In particular, we have shown in [32] that the network of synthetic genetic oscillators which we investigate here is characterized with inherent multistability and multirhythmicity, due to the inhibitory, phase-repulsive coupling which is present. Moreover, the dynamics of the genetic network gives further insight into its functional properties. Thus, in order to investigate in detail the dynamics of the coupled system under stochastic influence, we restrict initially our stochastic bifurcation analysis on the minimal case of 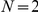 coupled cells. Finally, we generalize the obtained results on a illustrative example of 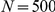 coupled cells.

**Figure 7:**
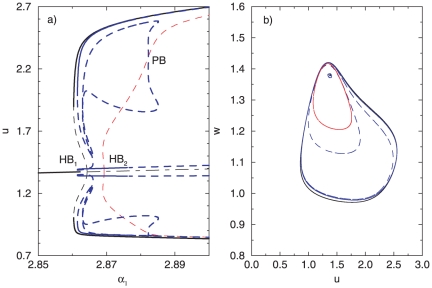
Characteristics of two coupled oscillators (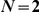) in the deterministic case (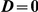) close to 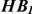 and 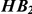. (A) A fragment of the bifurcation diagram. Here and in the following charts, the black lines stand for the in-phase oscillations, red for the anti-phase and blue for the asymmetric oscillations. Solid lines correspond to stable solutions, dashed lines for unstable, and dash-dotted line indicates the unstable focus. (B) Phase portrait for the multystability region at 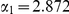. Parameters: 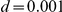, 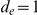.

One of the characteristic features of the coupled cellular system is the presence of two separate Hopf bifurcations, extremely close to each other in the parameter space (in Fig. 7a, 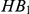 is located at 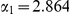 and 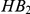 at 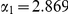). The Hopf bifurcations 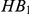 and 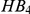 give rise to a branch of periodic orbits, corresponding to a synchronous in-phase solution, whereas 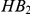 and 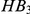 give rise to anti-phase solution. Additionally, a secondary bifurcation structure appears through a pitchfork bifurcation (labeled PB in Fig. 7a) on the anti-phase branch, resulting in a stable asymmetric regime. This regime is characterized with the presence of large and small amplitude oscillations in one attractor (for a detailed explanation of the dynamical regimes, see [32]). Due to the complexity of the bifurcation structure of the system, and in oder to investigate the stochastic transitions more accurately, we divide the characteristic 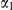 parameter interval in two separate parts, 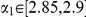 and 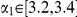.

For a fixed parameter (from the first interval) 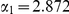, e.g., the coupled system displays a complex multistable structure, including an unstable focus, a stable in-phase cycle, an unstable anti-phase cycle, stable asymmetric branches (of the small and large amplitude oscillations) and an unstable cycle from the asymmetric branch (Fig. 7b).

**Figure 8:**
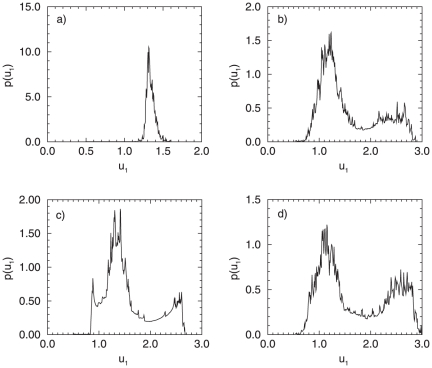
Probability distributions for a system of two coupled oscillators (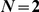) in the presence of noise. (A) 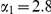, 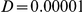; (B) 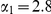, 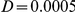; (C) 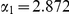, 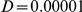; (D) 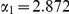, 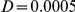.

In the presence of noise, however, a one-to-one correspondence between the deterministic and the stochastic attractors can not be established, since under noise, the lifetime of the attractors is rather short, or they merge. Therefore, it is appropriate to investigate the dynamical changes in the coupled system from the aspect of transformations of the distribution of the phase variable in terms of phenomenological stochastic bifurcations. We demonstrate here two separate cases: *i)* the system is located left to the tangent bifurcation, 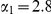, and in the deterministic case only the focus is stable, and *ii)* 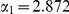, right after the 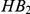, where the coexistence of five separate attractors is present, two of them are stable (the attractor of the in-phase oscillations and the attractor of the asymmetric oscillations, which is manifested via two separate stable branches - one corresponding to small, and one to large amplitude oscillations). For 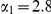 and a small noise intensity (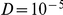), the trajectory naturally spends most of the time in the vicinity of the focus and the resulting distribution has one maximum (Fig. 8a). This means that under very small amount of noise, the genetic network produces rather constant protein concentrations, determined by the peak of the probability distribution. An increase of the noise intensity 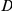, however, leads to more frequent visits of the trajectory to the region far away from the origin, inducing oscillations in the vicinity of the stable cycles which exist here. Again, a stochastic 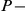
bifurcation occurs: a transition from a unimodal (for 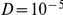) to a bimodal distribution (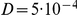) (Fig. 8b). We can state that the increase in 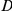 influences the dynamical behavior of the genetic network, manifested through changes in the probability for synthesis of a given protein, the 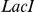 in this case. The system has now a complex trajectory, resulting in a possibility that the genetic network expresses different 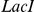 concentration levels, manifested through peaks in the corresponding probability distribution. For 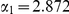 however, due to the presence of six separate branches (in the deterministic case), even for small noise intensities, i.e. of the order 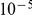, a clear multipeak distribution is manifested for the protein concentration of the observed gene (Fig. 8c). The positioning of the peaks in the distribution resembles the stable attractors in the deterministic case: the middle peak of the distribution, e.g., in Fig. 8c corresponds to the positioning of the focus and the stable branch of the small amplitude oscillations. Thus this peak is more pronounced in the corresponding distribution. For increased noise intensity (i.e. 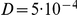) however, we can not establish any longer direct correspondence to the deterministic attractors. The trajectory which the system performs in the phase plane is again complex, and further leads to the disappearance of the middle peak in the distribution, characteristic for 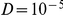. Thus, a clear, bimodal distribution emerges (Fig. 8d).

**Figure 9:**
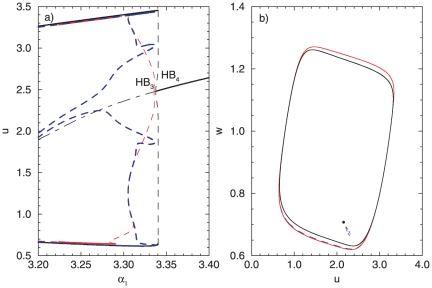
Characteristics of two coupled oscillators (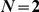) in the deterministic case (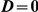) close to the 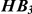 and 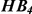. (A) A fragment of the bifurcation diagram. (B) Phase portrait for the multystability region 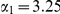.

For large 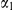 values the positioning and the number of stable attractors in the deterministic case changes. Namely, for 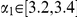 the asymmetric oscillations loose, whereas the anti-phase solution gains its stability. The characteristic bifurcation diagram is shown in Fig. 9a, and the corresponding phase plane view for 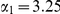 in Fig. 9b. Hence, for 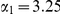, four separate attractors exist, two of them are stable: one characterizing the in-phase and the other one the anti-phase oscillations.

**Figure 10:**
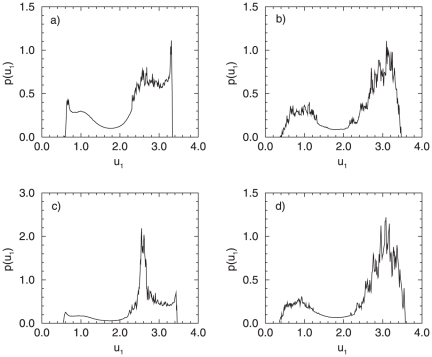
Probability distributions for a system of two coupled oscillators (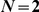) in the vicinity of 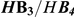. (A) 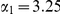, 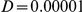; (B) 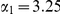, 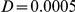; (C) 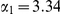, 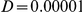; (D) 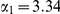, 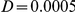.

Although the region of stability of the anti-phase oscillators is small, for small noise intensities (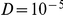) the peaks of the probability distributions can be related to the deterministic positions of the present attractors which are stable. One could speculate that since both stable attractors are located very close to each other in the phase plane (Fig. 9b), the noise leads to the appearance of plateaus in the probability distribution (Fig. 10a). The peaks are now wide, which results in larger interval values where the concentration of the expressed protein can be found. In contrast to the case for 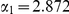, the peak at the middle of the probability distribution is not observed here. For the increased noise intensity (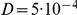), we observe a clear bimodal distribution (Fig. 10b). For 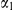 values exactly after the 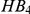 (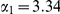), even small noise intensity (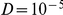) helps to pronounce the peaks corresponding to the deterministic cycle. Thus, the peaks, each located to low respectively high 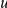 values are characteristic for the corresponding probability distribution, as shown in Fig. 10c. A stochastic 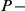
bifurcation is observed then for a noise intensity 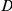 of order 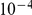, manifested through a transition to a bimodal distribution. The probability that a given protein concentration will be expressed in the genetic network is now restricted to two separate concentration intervals, one for low and one for high protein values.

Following the investigations presented here, we have seen that the dynamics of a particular network significantly varies when switching from deterministic to stochastic systems. The latter ones are closer to natural situations. Hence, the expressed protein concentration levels in a given genetic network strongly depend not only on the biologically characteristic parameters, such as the expression strength of the genes (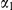), but also on the amount of noise 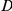 which is present in natural systems as well. The probability that a particular protein concentration is observed for small noise intensities could be related to the existence of deterministic attractors in the system, whereas for noise intensities of intermediate or larger strength, the probabilities are characetrized via stochastic bifurcations which describe the dynamics of the system.

However, the results for 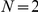 coupled cells do not differ significantly from the single unit example, despite the more complex bifurcation scenario present. The reason for this are the rather narrow parameter intervals where the additional attractors, which emerge for 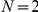, are stable. Thus, we observe the same type of stochastic 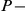
bifurcations.

**Figure 11:**
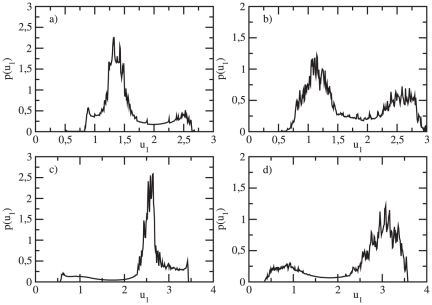
Probability distributions for a system of 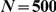 oscillators in the presence of noise. (A) 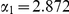, 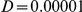; (B) 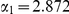, 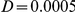; (C) 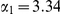, 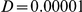; (D) 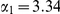, 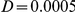.

The results presented for the case of 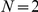 coupled cells can be easily generalized to larger networks, since they reflect the dynamical properties of networks of any size. We therefore investigate next the stochastic bifurcation structure of a genetic network consisting of 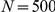 separate cells, and analyze the stochastic behavior both, when the parameter 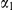 is in the vicinity of 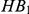 and 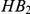, but also for 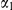 close to 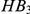. Fig. 11 shows that on both sides of the 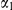 parameter interval, changes in the dynamical structure of the system can be captured through stochastic bifurcations, when varying the noise intensity 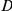.

The changes in the probability distribution which we observe for various noise intensities reflect the changes in the dynamical properties of the genetic network. Due to the stochastic influence, the expression of given proteins can be confined in several different concentration intervals, some of which are more probable than the others, depending how strong is the noise in the system. On the other hand, different protein concentrations in identical cells mean also different cellular functionality. This allows us to speculate that the differentiation which occurs in initially identical cellular populations is a result of a complex interplay between the intercellular signaling mechanisms which determines the dynamical structure of the network on one side, and the stochasticity characteristic for the particular system on the other. One could then successfully track the corresponding dynamical transitions by means of stochastic bifurcations, and address the question how cellular diversity evolves in relation to inherent stochasticity.

## Discussion

The dynamical behavior of natural genetic networks is influenced by two main factors: *i)* presence of internal stochasticity and *ii)* external factors, such as inputs from (co)regulating signaling networks. In this work we have defined a notion of stochastic bifurcation structures suitable for studying the behavior of genetic networks under stochastic conditions, covering both of the previously mentioned cases. A careful quantitative estimation of the stochastic bifurcation structure of (in this case, using the notion of reduced complexity, synthetic) genetic networks facilitates understanding of various natural phenomena, e.g. how cellular diversity evolves in relation with stochasticity and intercellular dynamics. In particular, we have shown that under stochastic influence, the behavior, even of a single cell, can not be directly related to the number and position of the stable deterministic attractors. However, the changes which occur in the probability distribution of a phase variable for e.g., could be used to track the dynamical transitions when the noise in the system varies. Additionally, the presence of noise could be regarded as a “survival factor”: we have shown that for intermediate noise intensity, the cell exhibits most coherent dynamical behavior, adapting the production of the corresponding proteins of interest to specific intervals of concentration values, most profitable for the cell. Considering next the interplay between stochasticity and intercellular signaling mechanisms, we have shown that genetic networks of any size could preform various coherent dynamical behavior, with proteins expressed in defined concentration intervals for different noise intensities. This means that under changing stochastic conditions, the probabilities of expressing certain concentration values are different, leading to different functionality of the cells, and thus differentiation of the cells in various types. The switching between various cell types is further determined by the peaks of the probability distribution showing that identical units can express proteins with various concentration values on one hand, and the noise intensity which determines, on the other hand, the shape of the probability distribution for the corresponding variable. Moreover, we have shown that external factors, such as regulatory networks which determine the expression of a given gene, strongly influence the produced protein in the system, by modifying the frequency with which the protein will be expressed. This characetristic of the network could be additionally used to control externally the timing of protein expression, which could further lead to optimization of various biological processes. As a prospect, it would be specifically intersting to study how cellular diversity is developed under stochasticity in growing populations, using the concept of stochastic bifurcations to follow the dynamical changes which occur correspondingly in the genetic networks.

In our studies we consider the global homogeneous coupling that can be easily implemented experimentally. As a future prospective it could be interesting to study the case where the cells are not exposed to identical environment, although the anisotropic coupling is extremely difficult to realize in a real experiment. This question needs a special investigation that can be addressed in future.
